# Preliminary analysis of the sinusoidal endothelium and space of Disse in ageing *Papio hamadrayas*

**DOI:** 10.1186/1476-5926-2-S1-S26

**Published:** 2004-01-14

**Authors:** Victoria C Cogger, Alessandra Warren, Robin Fraser, Meng Ngu, Allan J McLean, David G Le Couteur

**Affiliations:** 1 Centre for Education and Research on Ageing and ANZAC Research Institute, University of Sydney, Concord RG Hospital, Sydney, Australia; 2 Department of Pathology, Christchurch School of Medicine and Health Sciences, University of Otago, Christchurch, New Zealand; 3 Department of Gastroenterology, Concord RG Hospital, Sydney, Australia; 4 National Ageing Research Institute, Parkville, Melbourne, Australia

## Introduction

In the past it has been reported that the liver is free of any major age-related morphological changes apart from binucleate hepatocytes and lipofuscin deposition. Diminished hepatic blood flow and mass have been considered to be responsible for the decreased metabolic capacity seen in old age [1]. Recently we studied the ageing rat liver [2] and the ageing human liver [3] and found that there are a number of age-related changes to the perisinusoidal region at the ultrastructural level.

**Figure 1:**
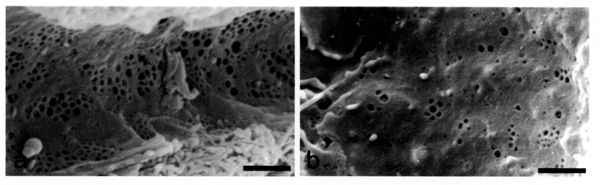
Scanning electron micrographs taken from rats aged 4 months (a) and 24 months (b). The aged endothelium is defenestrated. Bar = 1 –m.

These changes are called pseudocapillarization and are characterized by defenestration and thickening of the endothelium, basal lamina deposition and extracellular matrix deposition in the space of Disse (Figure 1). Expression of von Willebrands factor in the sinusoidal cells in old rats was found together with increased perisinusoidal expression of collagen. In this preliminary study we have examined the livers of young and old baboons to confirm that pseudocapillarization is a generalized ageing change across species.

## Methods

The animals used in this study were from a captive breeding colony of *Papio hamadrayas*. The experimental protocol was approved by the Central Sydney Area Health Service animal welfare committee. Liver tissue was collected by needle biopsy or from fresh post mortem animals. Half of each specimen was fixed for light microscopy and immunohistochemistry in 4% buffered paraformaldehyde and the other half was fixed for electron microscopy with 2% gluteraldehyde/ 3% paraformaldehyde in 0.1 M sodium-cacodylate buffer (0.1 M Sucrose, 2 mM CaCl_2_). Specimens fixed for light microscopy and immunohistochemistry were embedded in paraffin blocks. Sections from each animal were stained with H & E, Masson's trichrome and Wilder's reticulin. Immunohistochemistry was also used to detect the expression of Collagen IV, Laminin, Synaptophysin, Fibronectin, von Willebrands factor (vWF) and CD68. Tissue for transmission and scanning electron microscopy was prepared by standard methods [4] and randomly sampled for examination. Transmission electron micrographs were taken at 17 000– magnification (Philips CM120) and scanning electron micrographs were taken at 40 000– magnification (Philips XL30).

## Results

**Figure 2:**
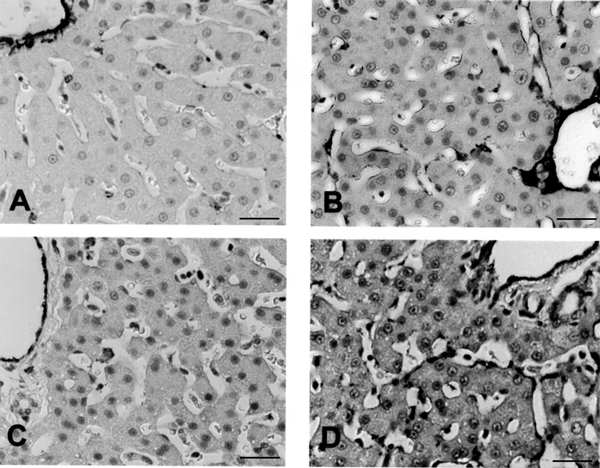
Immunohistochemistry studies for Laminin ([A] young baboon, [B] old baboon) and von Willebrands factor ([C] young baboon, [D] old baboon). There is increased perisinusoidal expression of both these antigens in the livers of old baboons. Bar = 20 –m.

All specimens were examined by a hepatopathologist to exclude recognized disease or pathology. Three young (6–7 years) and five old (20–24 years) baboons have been included in this study. On light microscopy no major differences were seen between the young and old baboons. On immunohistochemistry there was a slight increase in perisinusoidal expression of vWF and Laminin in the aged animals (Figure 2).

**Figure 3:**
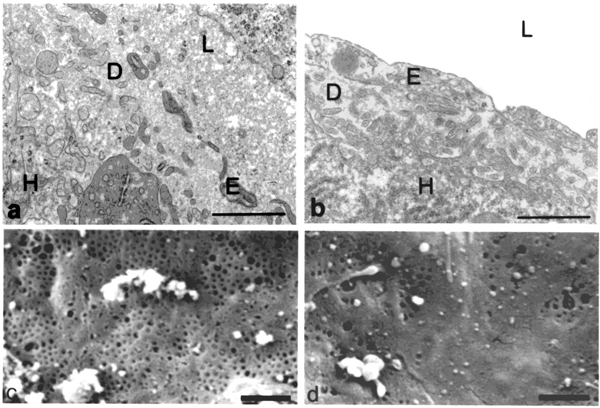
Transmission and scanning electron micrographs of baboon livers. In the young baboon (a & c) the endothelium (E) is thin & fenestrated and the space of Disse (D) is free of fibrosis. In the old baboon (b & d) the endothelium is thickened & defenestrated. H and L indicate the hepatocyte and sinusoidal lumen, respectively. Bar = 1 –m.

On electron microscopy a number of changes were noted. The endothelium was thicker and more continuous, with reduced numbers of fenestrations and decreased porosity (Figure 3).

## Discussion

The findings confirm that significant age-related changes occur in the perisinusoidal region of the baboon liver. These changes are similar to those we have previously reported in rats and humans which suggests that pseudocapillarization is a widespread ageing change. Defenestration of the endothelium is associated with increased plasma lipids and may explain the association of old age with atherosclerosis [5], an other diseases potentially linked to impaired hepatic function.
